# Pseudotumoral Pulmonary *Mycobacterium avium* Disease in a Patient on Ruxolitinib Therapy

**DOI:** 10.3390/diagnostics16071069

**Published:** 2026-04-02

**Authors:** Ancuta-Alina Constantin, Ana-Luiza Iorga, Andreea-Dumitrita Gaburici, Iustina Leonte

**Affiliations:** 1 Department of Cardio-Thoracic Pathology, “Carol Davila” University of Medicine and Pharmacy, 050474 Bucharest, Romania; ancuta-alina.constantin@umfcd.ro; 2 Institute of Pneumology “Marius Nasta”, 050159 Bucharest, Romania; analuiza_iorga@yahoo.com

**Keywords:** *Mycobacterium avium*, ruxolitinib, lung cancer mimicker, JAK inhibitors

## Abstract

Pulmonary disease caused by nontuberculous mycobacteria represents an important diagnostic challenge, particularly in immunocompromised patients, in whom clinical and radiologic findings may mimic malignancy. We report the case of a 70-year-old woman with myelofibrosis treated with ruxolitinib who developed a tumor-like lesion in the left upper lobe on computed tomography, highly suggestive of lung cancer. Despite broad-spectrum antibiotic therapy, the lesion persisted; bronchoscopy did not yield diagnostic findings, and CT-guided transthoracic biopsy demonstrated necrotizing granulomatous inflammation without evidence of malignancy. Microbiological analysis subsequently identified *Mycobacterium avium*, and targeted antimycobacterial therapy led to clinical and radiologic improvement. This case highlights that pulmonary nontuberculous mycobacterial infection may present as a pseudotumoral lesion and should be considered in the differential diagnosis of mass-like pulmonary opacities, particularly in patients receiving Janus kinase inhibitor therapy.

**Figure 1:**
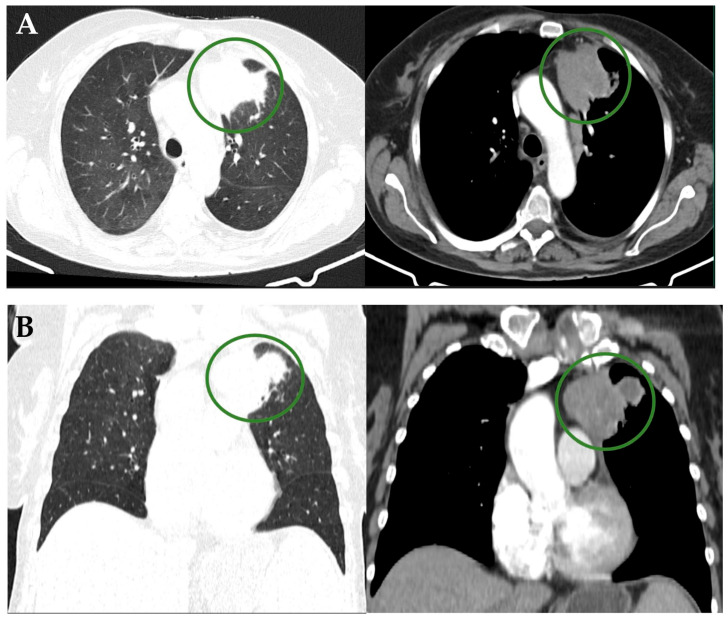
(**A**,**B**) Axial and coronal thoracic computed tomography (CT) findings in a 70-year-old woman showing a 1.9 × 1.1 cm nodule located in the anterior segment of the left upper lobe highlighted by green circles, showing heterogeneous attenuation, minimal peripheral calcifications, peripheral contrast enhancement, and central necrosis, with irregular margins and contact with the parietal pleura. Overall, the imaging features are suggestive of an inflammatory process; however, the presence of an associated tumoral mass cannot be excluded. Associated findings include near-complete atelectasis of the anterior segment of the left upper lobe and lamellar atelectases in the lingular segment. Multiple micronodules and small nodules are scattered throughout both lungs, some subpleural, measuring up to 4 mm. Medical history revealed multiple cardiovascular comorbidities and myeloid metaplasia with myelofibrosis, for which treatment with ruxolitinib was initiated in May 2023. The patient reported an insidious onset of symptoms: exertional dyspnea (mMRC score 3), profuse night sweats, and marked physical asthenia. On physical examination, there were decreased vesicular breath sounds at the left basal hemithorax and an oxygen saturation of 88% on room air. Blood tests demonstrated a significant inflammatory response (ESR 66 mm/h, CRP 1.91 mg/dL), leukocytosis (20,080/μL) with neutrophilia (14,680/μL), and mild iron-deficiency anemia (Hb 10.2 g/dL), abnormalities that could be attributed to the previously mentioned neoplastic comorbidities. To further clarify the diagnosis, the patient underwent flexible bronchoscopy, which showed no endobronchial lesions on examination using both white light and autofluorescence. A bronchial aspirate from the left upper lobe was collected for routine bacterial culture and acid-fast bacilli staining. Given the lack of clinical and laboratory improvement following broad-spectrum antibiotic therapy (initially ceftriaxone 1 g every 12 h, subsequently escalated to meropenem 1 g every 8 h) and the absence of microscopic or bacteriological abnormalities in the samples collected during bronchoscopy, a CT-guided transthoracic lung biopsy was performed.

**Figure 2:**
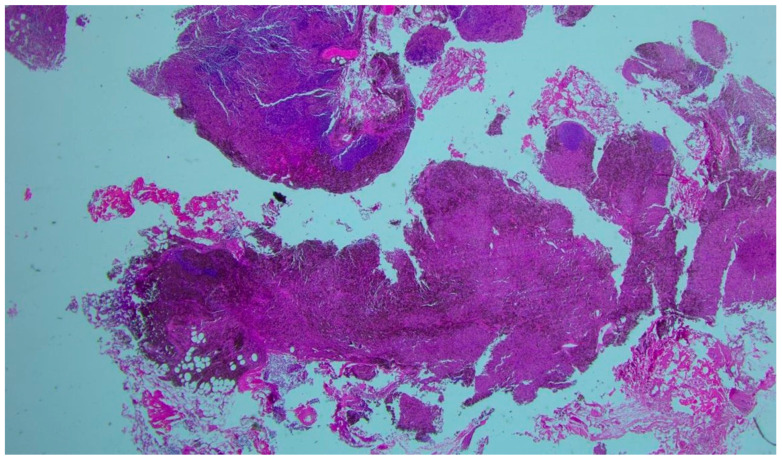
Histopathological examination (hematoxylin and eosin stain, original magnification: ×250) revealed lung parenchyma with a dense granulomatous lesion characterized by confluent nodules of histiocytes on a background of lymphocytes and black anthracotic pigment. The specimen showed multinucleated giant epithelioid cells of variable size, areas of suppurative necrosis, and bronchial metaplasia of the alveolar epithelium, with no evidence of malignant cells. Immunohistochemical analysis demonstrated CD68-positive histiocytes within granulomas and a mixed lymphocytic infiltrate (CD3 and CD20 positive). Cytokeratin AE1/AE3 staining highlighted only residual alveolar epithelium, with no evidence of epithelial malignancy. These findings supported a necrotizing granulomatous inflammatory process rather than a neoplastic lesion. At this point, the differential diagnosis was broadened to include infectious and inflammatory diseases, such as tuberculosis, nontuberculous mycobacterial infections, fungal infections, sarcoidosis, and granulomatosis with polyangiitis. Although the initial bronchial aspirate results appeared within normal limits, the cultures on Lowenstein–Jensen medium turned positive for atypical mycobacteria, with identification of *Mycobacterium avium* using a line probe assay (LPA). Given the persistence of symptoms in an immunocompromised patient, antimycobacterial therapy was initiated, consisting of rifampicin (450 mg), ethambutol (800 mg), clarithromycin (1000 mg), and moxifloxacin (400 mg). The treatment was well tolerated, and the patient showed marked clinical improvement during follow-up. At 11 months after initiation of therapy, the patient remains in good general condition, with no clinical signs suggestive of malignancy and with partial radiologic regression (Figure 3).

**Figure 3:**
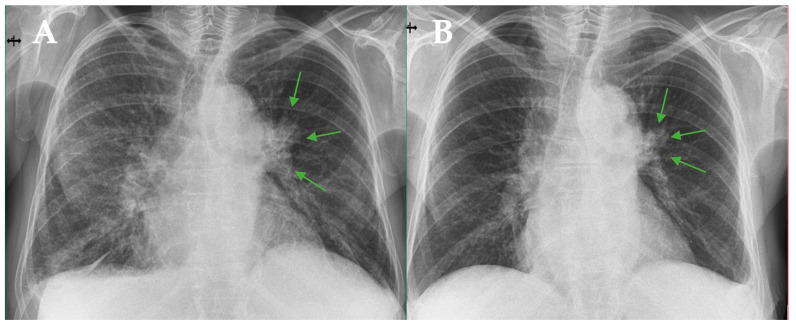
Posteroanterior chest radiographs. (**A**) At the initial evaluation. (**B**) Ten months after treatment initiation, showing a slight reduction in the size of the left hilar lesion, indicated by green arows. Immunosuppression is a major risk factor for various infections, including nontuberculous mycobacterial (NTM) infection. Patients with myelofibrosis treated with ruxolitinib are particularly vulnerable to mycobacterial infections due to impaired cellular immunity [1,2]. Ruxolitinib, a JAK1/2 inhibitor, has been associated with opportunistic infections, including tuberculosis and nontuberculous mycobacterial disease [2]. The increased susceptibility is thought to be related to modulation of T cell-mediated immune responses, particularly reduced regulatory and effector T cell function [3]. The distinctive feature of this case is the pseudotumoral radiologic appearance of *Mycobacterium avium* infection. Although MAC pulmonary disease typically presents with nodular bronchiectatic or cavitary patterns, mass-like consolidation has rarely been described and may mimic lung malignancy [4]. In immunocompromised hosts, altered granulomatous response and reduced necrosis may contribute to this atypical imaging pattern. A similar association between ruxolitinib therapy and atypical pulmonary infections has been previously reported [5]. This case highlights the importance of including NTM infection in the differential diagnosis of tumor-like pulmonary lesions in patients receiving JAK inhibitors, in order to avoid misdiagnosis and inappropriate oncologic management.

## Data Availability

No new data were created or analyzed in this study. Data sharing is not applicable to this article.
